# Combination of eribulin and anlotinib exerts synergistic cytotoxicity in retroperitoneal liposarcoma by inducing endoplasmic reticulum stress

**DOI:** 10.1038/s41420-024-02103-2

**Published:** 2024-08-08

**Authors:** Shuquan Li, Hongtao Zhang, Hao Yu, Yifan Wu, Liang Yan, Xiaoya Guan, Bin Dong, Min Zhao, Xiuyun Tian, Chunyi Hao, Jianhui Wu

**Affiliations:** 1 Key Laboratory of Carcinogenesis and Translational Research (Ministry of Education), Department of Hepato-Pancreato-Biliary Surgery, Peking University Cancer Hospital & Institute, Beijing, China; 2 Guowen (Changchun) International Hospital, Changchun, Jilin Province China; 3 Key Laboratory of Carcinogenesis and Translational Research (Ministry of Education), Central Laboratory, Peking University Cancer Hospital & Institute, Beijing, China; 4 Key Laboratory of Carcinogenesis and Translational Research (Ministry of Education), Department of Pathology, Peking University Cancer Hospital & Institute, Beijing, China

**Keywords:** Translational research, Sarcoma, Sarcoma

## Abstract

Primary retroperitoneal liposarcoma (RLPS) is a rare heterogeneous tumor occurring within retroperitoneal space, and its overall survival has not improved much in the past few decades. Based on a small-sample clinical practice at our center, patients with RLPS can greatly benefit from anlotinib and eribulin combination. In this study, we investigated the combinational effect of anlotinib and eribulin on RLPS. In vitro experiments revealed that a low dose of anlotinib significantly enhances the cytotoxic effects of eribulin, leading to a remarkable suppression of RLPS cell proliferation, viability, colony formation, migration, and cell-cycle progression compared to individual drug treatments. At the organoid level, the combination treatment causes the spheroids in Matrigel to disintegrate earlier than the single-drug group. In vivo, RLPS patient-derived xenograft (PDX) models demonstrated that the combination of these two drugs can obviously exert a safe and effective anti-tumor effect. Through transcriptome analysis, we uncovered and validated that the synergistic effect mainly is induced by the endoplasmic reticulum stress (ERS) pathway both in vitro and in vivo. Further analyses indicate that anlotinib plus eribulin treatment results in micro-vessel density and PD-L1 expression alterations, suggesting a potential impact on the tumor microenvironment. This study extensively explored the combination regimen at multiple levels and its underlying molecular mechanism in RLPS, thus providing a foundation for translational medicine research.

## Introduction

Retroperitoneal liposarcoma (RLPS) stands out as the most prevalent sarcoma originating within the retroperitoneal space [1]. Based on morphology and genetic characteristics, the WHO classified liposarcoma into 5 pathological subtypes, and the most abundant subtypes are well-differentiated liposarcoma (WDLPS) and de-differentiated liposarcoma (DDLPS) [2, 3]. Due to its inert biological profile, RLPS is generally considered to be relatively insensitive to chemotherapy and radiotherapy [4, 5]. Currently, surgical excision remains the most effective curative treatment, however, local recurrence is still common and responsible for most mortality [6, 7]. Patients ineligible for surgery face limited options, thus it is urgently needed to identify innovative systemic methods to improve RLPS outcome.

Doxorubicin-based anthracycline drugs have been the first line of chemotherapy for local advanced or metastatic soft tissue sarcoma for decades [8], while overall results are still unsatisfying. Recently, trabectedin and eribulin have been introduced to treat soft tissue sarcomas, which brings new opportunities for RLPS. Eribulin is a newly developed microtubule inhibitor derived from halichondrin B which was naturally discovered in marine sponges [9]. Distinct from other microtubule inhibitory agents, eribulin inhibits only the growth phase of the microtubule and prevents mitotic spindle assembly during prometaphase [10]. Pre-clinical studies showed that eribulin can also reverse epithelial–mesenchymal transition [11] and modulate intra-tumoral vascular circulation in breast cancer [12]. A randomized phase III trial has revealed that eribulin prolonged the overall survival of liposarcoma patients compared to dacarbazine, and eribulin has been further approved for treating unresectable or metastatic liposarcoma [13].

Apart from conventional surgery and chemotherapy treatment, targeted therapies are also being actively developed. However, targeted therapy options for RLPS remain relatively limited, with pazopanib being the only agent approved for treating soft tissue sarcoma (excluding GIST) in the US. Unfortunately, pazopanib has disappointing effectiveness for adipocytic malignancy based on a phase II clinical trial, positioning it merely as a safer alternative [14, 15]. Anlotinib (AL3818) is a novel multi-target tyrosine kinase inhibitor (TKI) targeting VEGFR1-3, FGFR1-4, PDGFR-α, PDGFR-β, and c-kit. It has both effects of anti-angiogenesis and direct inhibition of tumor growth [16]. In vitro, anlotinib has been demonstrated superior anti-angiogenesis effects compared to sunitinib, sorafenib, and nintedanib [17]. Other pre-clinical models have shown its anti-tumor effects in non-small cell cancer lung cancer [18], thyroid cancer [19], intrahepatic cholangiocarcinoma [20], pancreatic cancer [21] and soft tissue sarcoma [22]. A randomized, double-blind, multi-center, phase II clinical trial enrolled 233 patients with advanced soft tissue sarcoma, and the study found that the median progression-free survival (mPFS) in the anlotinib group was extended by 4.8 months compared with the placebo group [23]. The objective response rate (ORR) and the disease control rate for anlotinib were also improved in this study. Due to its unique targets to vessel and mesenchymal-derived cells, anlotinib is a promising TKI drug for RLPS patients.

Unlike other solid tumors, there is no consensus on a combination regimen for liposarcoma, and most combinations for advanced liposarcoma have not reached the expected efficacy in early clinical trials. Inspired by the LEADER study, eribulin plus TKI can exert promising efficacy in soft tissue sarcoma [24]. In our center, we found that patients with RLPS can greatly benefit from the combination of anlotinib and eribulin [25]. However, the combinational effect of the two drugs is not clear, and it is necessary to explore the specific molecular mechanism, which may provide a basis for clinical practice and further potential of this combination. In this study, we have extensively confirmed that anlotinib and eribulin, working in combination, have profound effects on RLPS in many ways, and this could bring new hope for RLPS patients.

## Results

### Patients with RLPS achieved regression after administration of anlotinib and eribulin combination

**Fig. 1:**
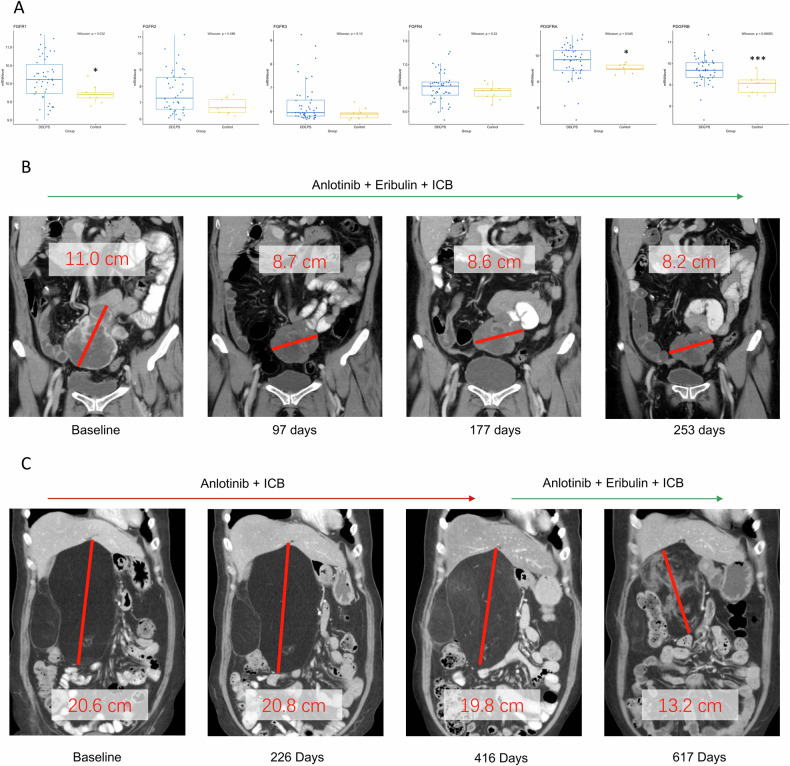
Bioinformatics analysis and clinical practice evidenced that RLPS patients could benefit from a combination of anlotinib and eribulin. **A** Major targets of anlotinib are upregulated in liposarcoma tissues (*n* = 46) compared to normal fat tissue (*n* = 9). Box plots were generated from the GSE21122 dataset, wherein mRNA level is presented in logarithmic scale. Statistical test: Wilcoxon rank test, *p < 0.05; **p < 0.01; ***p < 0.001. **B** A patient with DDLPS achieved partial regression after receiving the combination of anlotinib and eribulin. **C** A patient with WDLPS first achieved stable disease after anlotinib treatment and then achieved partial regression following eribulin co-administration. ICB immune checkpoint blockade.

To investigate if RLPS patients might benefit from additional anlotinib treatment, we compared the gene expression profile of 46 DDLPS and 9 normal adipose samples in the GSE21122 dataset (Fig. 1A). Most of anlotinib’s targets were upregulated in DDLPS, while FGFR1, PDGFRA and PDGFRB were significantly upregulated in tumor tissues. Our previous studies also identified VEGFR-2 and micro-vessel density (MVD) as key prognostic factors in RLPS [26]. Patients with recurrent RLPS who received combination therapy at our institution demonstrated favorable outcomes. A 59-year-old female patient (Fig. 1B) diagnosed with recurrent DDLPS received 9 cycles of anlotinib (12 mg, day 1–14, every 21 days) and eribulin (1.4 mg/m^2^, day 1 and 8) in combination with camrelizumab (200 mg, day 1), resulting in partial response. Another 56-year-old female patient (Fig. 1C) with recurrent well-differentiated liposarcoma (WDLPS) initially received 9 cycles of anlotinib (12 mg, day 1–14, every 21 days) with supplemental camrelizumab (200 mg, day 1), achieving stable disease. Subsequent co-administration of eribulin (1.4 mg/m^2^, day 1 and 8) led to a significant reduction in tumor volume. This evidence suggests the promise of this combination therapy, warranting further investigation into its underlying mechanisms.

### Establishment of primary DDLPS cell strains and IC_50_ determination

**Fig. 2:**
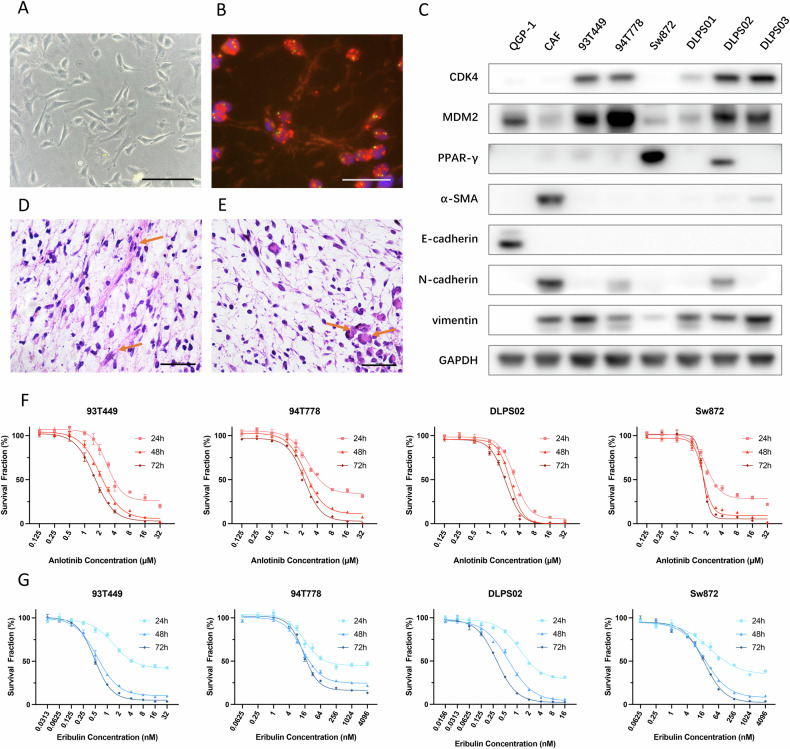
DDLPS cell strain validation and dose-response curve of RLPS cells. **A** Representative contrast microscopy image of the DDLPS cell strain (DLPS02) exhibiting spindle-like morphology. Scale bar: 200 μm. **B** FISH probe showing the DDLPS cell strain (DLPS02) exhibited MDM2 amplification. Scale bar: 20 μm (Red: MDM2 probe, Green: Cen12 control probe). **C** DDLPS cells were mesenchymal-derived (vimentin-positive) and accompanied by MDM2 and CDK4 expression while lacking epithelium marker (E-cadherin) and CAF marker (α-SMA). QGP-1 is a pancreatic tumor cell line, representing the epithelial cell profile. **D**, **E** HE staining of DLPS02 parental tumor and cell strain derived xenograft. Lipoblast-like cells (indicated with arrows) could be found, indicating their adipose origin. Scale bar: 50 μm. **F** 24, 48, and 72 h Anlotinib dose-response curve for 93T449, 94T778, DLPS02, and Sw872 cells. **G** 24, 48, and 72 h Eribulin dose-responsive curve for 93T449, 94T778, DLPS02, and Sw872 cells. Data from a representative experiment (*n* = 3 replicate cell cultures).

Since DDLPS cell lines are not commercially available for research, we established primary DDLPS cell cultures. In vitro, our DDLPS cell strains exhibited an adherent proliferation profile with minimal lipid deposits (Fig. 2A). DLPS02, derived from its parental DDLPS tumor, featured chromosome 12q13–q15 amplification with duplicated MDM2 gene (Fig. 2B). In Fig. 2C, western blot showed that our primary DDLPS cells overexpress CDK4 and are MDM2 positive, in line with specific 12q13–q15 amplification. Peroxisome proliferator-activated receptor gamma (PPAR-γ), the master regulator of adipocyte differentiation, is also found in some of these cells, indicating their adipose origin. In addition, the histological morphology of the DLPS02-derived xenograft resembled its parental tumor tissue, suggesting that the DLPS02 cell strain preserved its DDLPS characteristics (Fig. 2D, E). To evaluate the effect of anlotinib and eribulin on the proliferation of RLPS cells, DLPS02 together with other purchased RLPS cell lines (93T449, 94T778, and Sw872) were treated with anlotinib and eribulin at increasing concentrations for 24, 48 or 72 h. A CCK-8 assay was then carried out to measure cell viability. The inhibition of anlotinib and eribulin on each cell line showed dose- and time-dependent patterns (Fig. 2F, G), and respective IC_50_ values are listed in Table 1. In this study, we decided to use 48 h IC_50_ as the reference concentration for subsequent studies.

**Table 1:** The half maximal inhibitory concentration (IC_50_) of four RLPS cell lines.

		93T449	94T778	DLPS02	Sw872
Pathological subtype		Well-differentiated liposarcoma	Well-differentiated liposarcoma	De-differentiated liposarcoma	Undifferentiated or pleomorphic liposarcoma
Chr 12q13-15 amplification		Positive	Positive	Positive	Negative
Anlotinib IC_50_	24 h	3.31	2.89	2.93	2.42
(μmol/l)	48 h	2.16	2.38	2.32	1.86
	72 h	1.72	2.13	1.96	1.74
Eribulin IC_50_	24 h	1.36	19.35	0.95	31.23
(nmol/l)	48 h	0.72	13.32	0.51	18.96
	72 h	0.58	12.02	0.26	16.26

### Anlotinib synergize with eribulin in cytotoxicity in RLPS cells

**Fig. 3:**
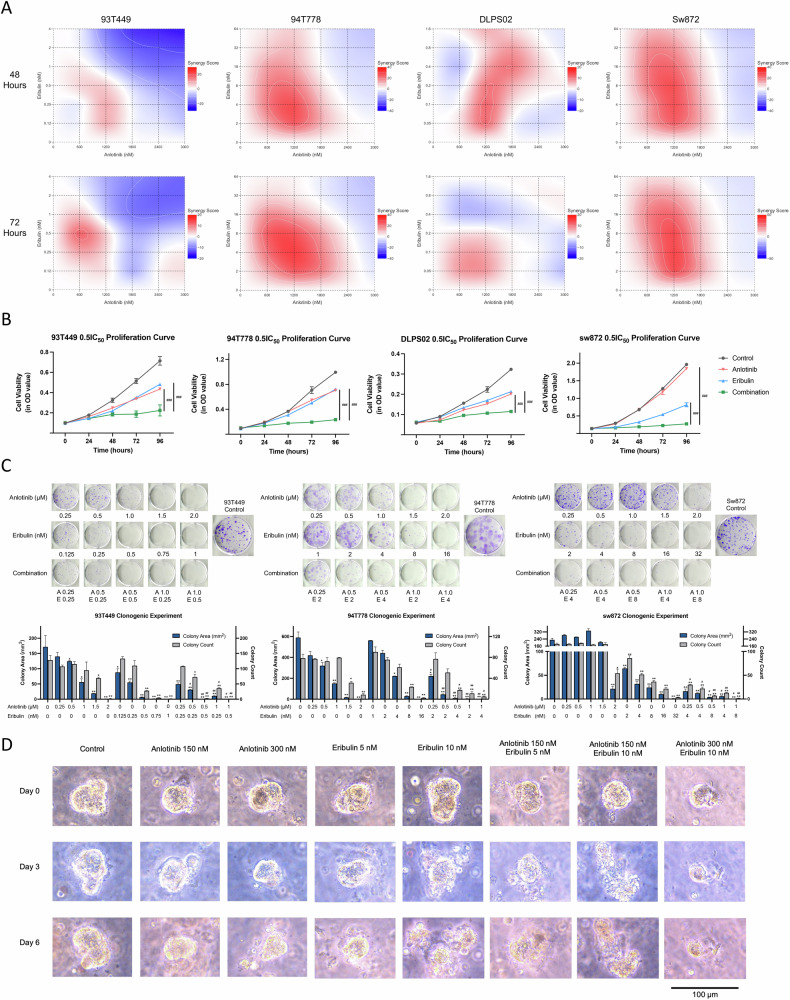
Anlotinib and eribulin had synergistic effects in RLPS. **A** Heatmap showed synergistic scores at different concentrations. Higher score (redder color) indicates synergy in proliferation inhibition, and a lower score (bluer color) suggests an antagonistic effect. **B** Proliferation curves of RLPS cell strains assessed by CCK-8. In single-drug groups, proliferation was significantly inhibited compared with the control group (not marked on the graph); and proliferation in combination groups was significantly inhibited compared to single-drug groups. Data from a representative experiment (*n* = 3, # compared to eribulin or anlotinib). **C** Colony formation assay showed combination treatment reduced colony formation and colony size (*n* = 2, * Compared to control; # compared to eribulin with corresponding concentration). **D** Representative RLPS contrast microscopy image of organoids showing spheroids were sensitive to combination therapy, especially at extremely low concentrations. */# *p* < 0.05; **/## *p* < 0.01; ***/### *p* < 0.001.

To determine if anlotinib and eribulin combination had synergistic effects, we tested various concentration matrices of the two drugs. As shown in Fig. 3A, these two drugs had a synergistic inhibition effect on cell proliferation, especially at lower concentrations. Based on the synergy hotspots in Fig. 3A, we decided to use half of IC_50_ concentration to validate this effect. The combination group showed a significant reduction in cell viability compared to the mono-drug treatment group (Fig. 3B). Colony formation assays were also performed to assess long-term inhibition, and we found that small doses of anlotinib plus eribulin significantly reduced both colony number and size (Fig. 3C). Finally, organoid models confirmed the superiority of the combination therapy over monotherapy. As shown in Fig. 3D, spheroids in the combination group started to collapse on day 3, while organoids in monotherapy groups broke down on day 6. Altogether, anlotinib demonstrated a remarkable synergistic effect when combined with eribulin.

### Combination of anlotinib and eribulin induced migration inhibition, apoptosis, and cell-cycle arrest in RLPS cells

**Fig. 4:**
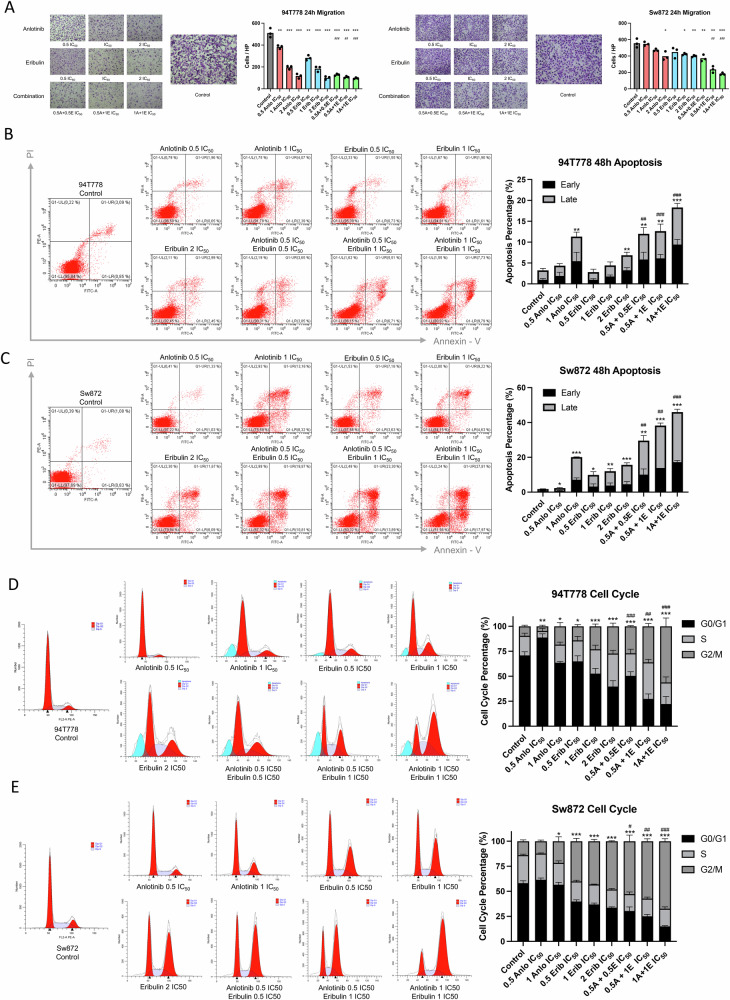
Combination of anlotinib and eribulin induces migration inhibition, apoptosis, and cell-cycle arrest. **A** Transwell assays revealed that anlotinib and eribulin combination hindered migration of RLPS cells (*n* = 3). Cells were counted per high-power field (HP). **B**, **C** Double stained flow-cytometry showed early and late apoptosis in 94T778 and Sw872. Statistical analysis includes all cells committing apoptosis (*n* = 3), and error bars representing SD for respective early and late apoptosis rates. **D**, **E** Combination therapy triggered 94T778 and Sw872 cell line G2/M phase arrest. Statistical analysis including G2/M phase percentage only (*n* = 3). * Compared to control; # compared to eribulin (with corresponding concentration). */# *p* < 0.05; **/## *p* < 0.01; ***/### *p* < 0.001.

We used Transwell assay to examine the migration capabilities of RLPS cells. Anlotinib or eribulin alone inhibited cells from passing through the chambers, whereas the combination regimen potently hindered cell migration compared to monotherapy (Fig. 4A). Next, whether the combination could induce apoptosis was determined. Both anlotinib and eribulin induced apoptosis in a concentration-dependent manner, while the combination group significantly boosted the apoptosis rate compared to eribulin alone (Fig. 4B, C). Particularly, at half of anlotinib IC_50_ concentration, apoptosis was barely evoked; however, in combination with eribulin, it boosted the apoptosis rate nearly 3-fold. Considering eribulin as a microtubule modulator, Fig. 4D, E reveals that low concentrations of eribulin resulted in G2/M cell-cycle arrest. Additionally, anlotinib also synergized with eribulin in inducing G2/M cell-cycle arrest compared to eribulin alone. In summary, the anlotinib and eribulin combination could effectively mitigate malignant behaviors of RLPS cells, and notably low concentration of anlotinib potentiated cytotoxicity of eribulin in a sensitizer manner.

### Synergistic effect of the combination was mainly induced by endoplasmic reticulum stress

**Fig. 5:**
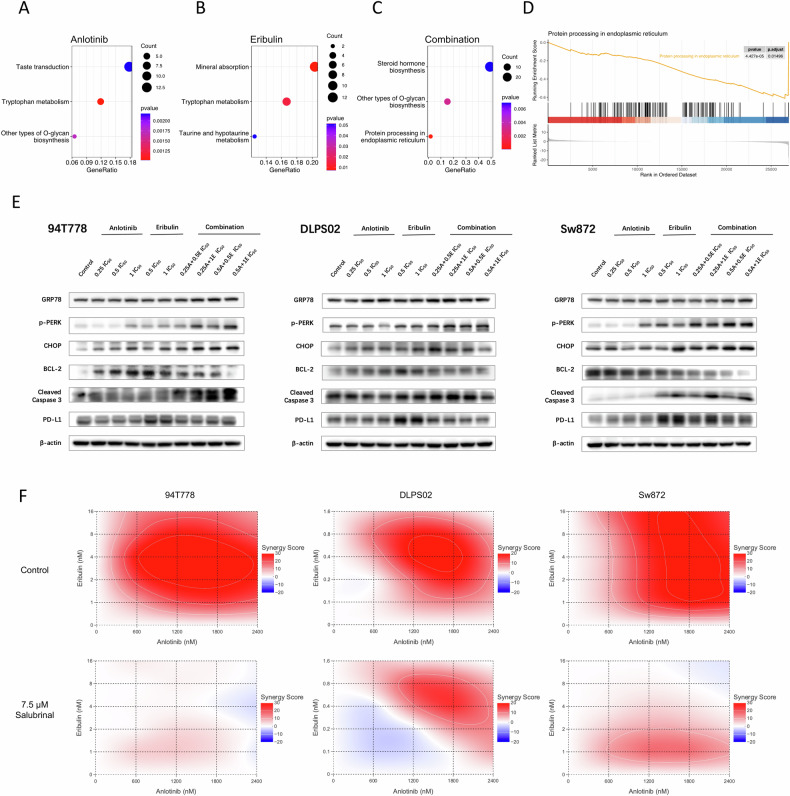
Synergistic effect of the combination is induced by endoplasmic reticulum stress. **A**–**C** Dot plots showing the top 3 KEGG pathway enrichment results of anlotinib, eribulin, and combination group. **D** GSEA plot featuring protein processing in endoplasmic reticulum pathway. **E** ERS induced by combination therapy was validated through western blot, wherein key ERS markers were upregulated and apoptosis pathway was initiated. **F** Rescue experiments further validated that a synergistic effect was largely induced by ERS. After applying salubrinal, synergy scores were significantly reduced.

To further elucidate the underlying mechanisms of the combination, RNA-seq was conducted to evaluate transcriptome changes in RLPS cells after being treated with the optimal concentrations that elicited maximum synergy effect. Global gene expression profile was changed after being treated with different drugs, and differentially expressed genes (DEG) were summarized in Supplementary Fig. 1 A–C. Through the GSEA algorithm and KEGG gene set analysis, we discovered that anlotinib altered RLPS in tryptophan and O-glycan metabolism; eribulin led to changes in mineral absorption and other amino acid metabolism, and the combination of anlotinib and eribulin introduced modulation in endoplasmic reticulum protein processing (Fig. 5A–C). Through intersecting altered pathways, we focused on the endoplasmic reticulum protein processing pathway which was significantly incited by combination therapy (*p*_adj_ = 0.015) (Fig. 5D). And further pathway mapping (Supplementary Fig. 1D) led us to focus on endoplasmic reticulum stress (ERS), which is a subcategory of protein processing in the endoplasmic reticulum. We performed western blots to validate whether the combination of two drugs could elicit ERS (Fig. 5E). GRP78, p-PERK, and CHOP are key proteins in the ERS pathway, which were upregulated in the combination group in response to unfolded protein and reactive oxygen species (ROS). Excess ERS would trigger downstream apoptosis reaction, resulting in BCL-2 degradation and caspase cascade activation (predominately caspase-3 cleavage). To further investigate the causal relationship between ERS and the synergistic effects, RLPS cells were treated with or without salubrinal, an inhibitor of ERS [27]. As shown in Fig. 5F, after 7.5 μM salubrinal pre-treatment, the 48-h synergistic effect of anlotinib and eribulin combination was largely diminished. These results suggested that anlotinib might synergize with eribulin in RLPS cell lines via inducing ERS. Previous literature stated that TKIs can modulate PD-L1 expression. We found that eribulin alone could lead to PD-L1 overexpression, while additional anlotinib treatment reversed PD-L1 upregulation induced by eribulin.

### Combination of anlotinib and eribulin inhibited the growth of RLPS in vivo

**Fig. 6:**
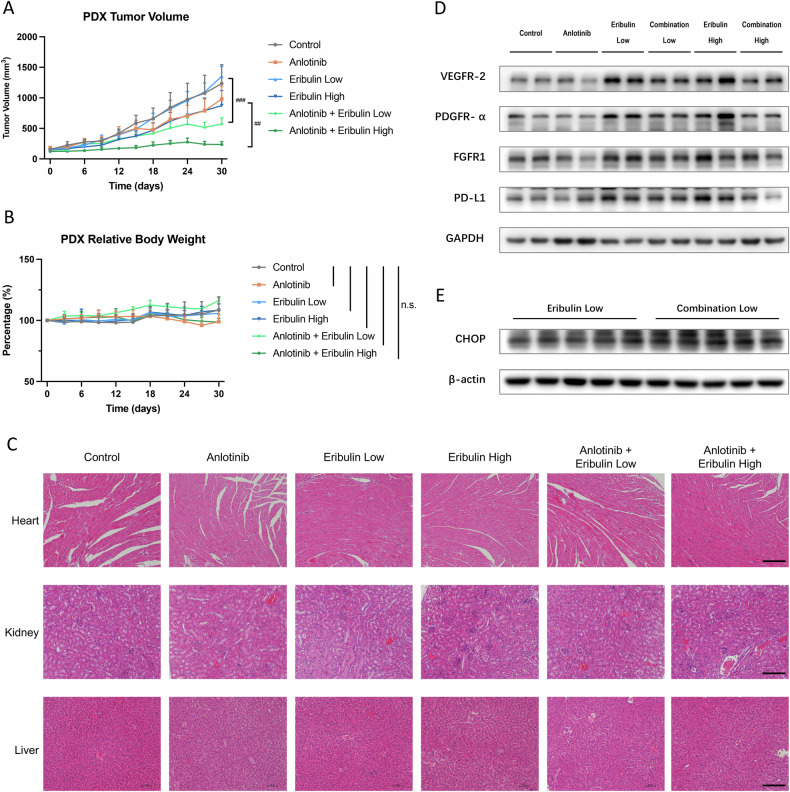
Combination of anlotinib and eribulin inhibited RLPS growth in vivo. **A** Tumor growth curves for anlotinib, eribulin, combination, and control group. Values represented as mean ± SEM (*n* = 5), # compared to eribulin group with corresponding concentration, ## *p* < 0.01; ### *p* < 0.001. **B** Body weight measurement for each group. n.s. non-significant. **C** Representative HE staining of major vital organs, no obvious changes can be observed. Scale bar: 200 μm. **D** Western blots demonstrating anlotinib diminished RTKs and PD-L1 overexpression induced by eribulin. **E** CHOP, as a critical ERS regulator, was upregulated in the combination group compared with the eribulin group.

We established RLPS patient-derived xenograft (PDX) to evaluate anlotinib and eribulin combinations in vivo. The PDX was stably passaged to P4 for subsequent experiments while retaining the original tumor characteristics. Anlotinib (1.5 mg/kg/day) was given orally and eribulin (Low dose: 0.25 mg/kg/week; High dose: 0.5 mg/kg/week) was administrated by intravenous injection. Mice were sacrificed after 30 days, and transplanted tumors were dissected (Supplementary Fig. 2). The monotherapy groups showed slower tumor growth on average compared to the control group, though the difference was not statistically significant. In contrast, the combination group significantly reduced tumor growth compared to the eribulin group (*p* = 0.003 and *p* = 0.036 for low and high eribulin doses, respectively) (Fig. 6A). For this drug combination, no obvious toxicity was observed at the applied doses, as indicated by the slight body weight change during the experiment (Fig. 6B). Further HE staining for heart, kidney and liver did not show apparent histological changes (Fig. 6C), suggesting that the combination regimen had limited cardiotoxicity, hepatotoxicity or nephrotoxicity. As a TKI drug, anlotinib has well-defined targets, so western blots were performed to assess how those targets changed upon co-administration with eribulin. Interestingly, VEGFR-2, PDGFR-α, and FGFR-1 were upregulated upon eribulin treatment, and the expression of these receptor tyrosine kinases (RTKs) was reduced in the combination group (Fig. 6D). Similar to what we previously found in cell lines, PD-L1 was upregulated upon eribulin treatment in vivo, and the addition of anlotinib reversed this PD-L1 overexpression. Furthermore, we also confirmed that CHOP was upregulated in combination with anlotinib and eribulin in vivo (Fig. 6E), indicating the synergistic effect of the regimen could also be provoked by ERS.

**Fig. 7:**
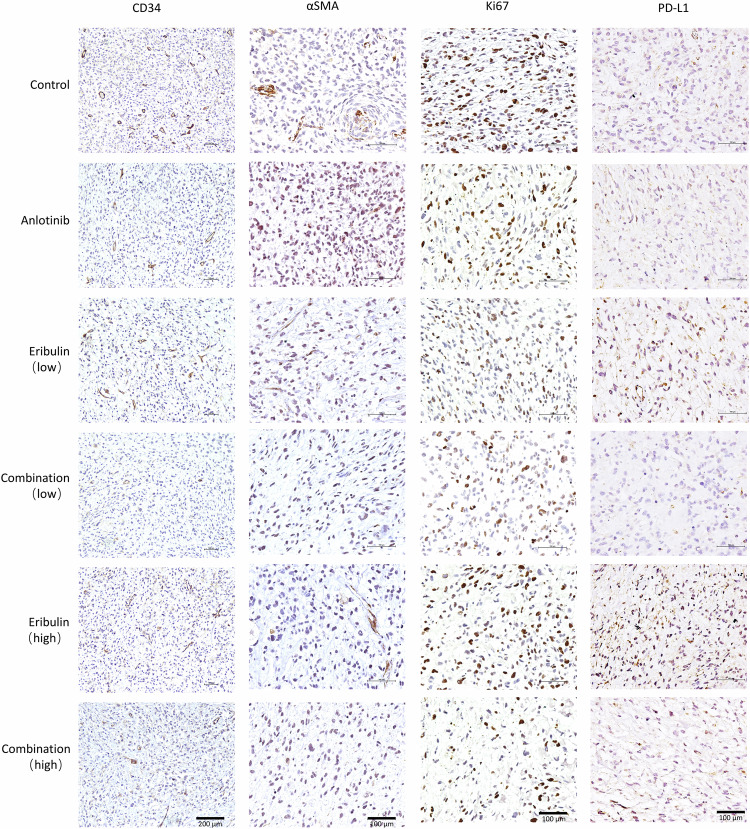
Monotherapy and combination modulated expression of CD34, α-SMA, Ki-67, and PD-L1 in RLPS PDX. CD34 labeled micro-vessels were decreased in the anlotinib and the combination group. α-SMA labeled CAFs were reduced in the combination group. Ki-67 is an indicator of proliferation, and it was reduced in the monotherapy group and further diminished in the combination group. PD-L1 was upregulated upon eribulin treatment, while a combination regimen reduced PD-L1 overexpression.

In addition, we used immunohistochemistry (IHC) staining to illustrate how the combination regimen interacted with RLPS, reflecting alterations in both tumor cells and other components (Fig. 7 and Supplementary Fig. 3). In particular, CD34 labeled MVD was reduced in both the anlotinib group and the combination group. Ki-67-positive cells were reduced in monotherapy groups and further decreased in combination groups. α-SMA marked cancer-associated fibroblasts (CAF) were also decreased in combination groups. PD-L1 was expressed across all groups, while eribulin-treated groups had more PD-L1-positive cells.

## Discussion

RLPS is a group of rare and heterogeneous malignancies with unique biological characteristics. Currently, surgical resection remains the only curative method, while systemic treatments for advanced and metastatic RLPS do not improve much. To address these unmet clinical needs, our center innovatively introduced a regimen combining anlotinib and eribulin. Based on our preliminary clinical practice results, we found that the combination achieved satisfying results in retroperitoneal soft tissue sarcoma with an ORR of 27.3% [25]. A recent phase II open-label clinical trial reported this combination prolonged median PFS and increased ORR in recurrent or metastatic breast cancer [28]. Another case report also suggested the combination of anlotinib and eribulin achieved a durable response in rare perianal adenoid cystic carcinoma [29]. Here, we explored for the first time the effect and underlying mechanisms of this combination in liposarcoma.

Since DDLPS cell lines are poorly reported and not commercially available, we established three primary DDLPS cell strains to represent this pathological subtype. Our cell strains carry the characteristic chromosome 12q13-15 amplification of DDLPS. MDM2 and CDK4, the predominant oncogenes in this region, are highly expressed in our cell strains, illustrating that they accurately preserved the biological profiles of DDLPS [30]. Along with other available cell lines, our research covered major RLPS pathological subtypes, including WDLPS, DDLPS, and pleomorphic liposarcoma. We also creatively utilized a mesenchymal stem cell-based medium to successfully build RLPS organoids. Together with PDX, we used multiple levels of the RLPS model for a more representative presentation in this study. In subsequent experiments, the combination of anlotinib and eribulin significantly inhibited the proliferation, migration, and cell cycle of liposarcoma cells compared to monotherapy. Moreover, we found that anlotinib and eribulin combination had a synergistic effect rather than a plain additive effect, indicating that the combination has greater efficacy at a lower dosage. Animal experiments also showed combination regimens had superior potency than single-drug treatment with no obvious side effects. Overall, our representative pre-clinical models validated the combination regimen has substantial anti-tumor effects both in vitro and in vivo.

To elucidate the underlying mechanism, transcriptome RNA-seq, and bioinformatics analysis were conducted, and we found the combination of anlotinib and eribulin eventually led to ERS. The endoplasmic reticulum is a central organelle responsible for maintaining protein anabolism and catabolism. When cells are subjected to intensive environmental stress or misfolded protein accumulation, the endoplasmic reticulum cavity will initiate series reactions known as ERS [31], and persistent stress will then result in the activation of the apoptosis cascade [31]. We proved combination regimen induced the upregulation of key ERS markers, including GRP78, p-PERK, and CHOP. Sustained ERS initiated apoptosis pathway, notably Bcl-2 downregulation, and caspase-3 cleavage, which has been confirmed by flow-cytometry and western blot. Another study reported that anlotinib alone could elicit ERS by introducing ROS [21], however, we noticed the concentrations they used were far higher than blood drug concentrations achievable in clinical settings [32]. Hence, we believe our combination regimen has more practical applications in clinical settings.

Acquired resistance is a major problem in the systemic treatment of tumor patients, and off-target resistance is commonly built after long-term chemotherapy [33–35]. Off-target resistance-related pathways are not directly against anti-tumor drugs but can compensate for the cytotoxic effects caused by chemotherapy. In this study, we observed that RTKs, promoting cell survival, were upregulated upon eribulin treatment. In accordance with other literature, soft tissue sarcoma develops off-target resistance through RTK upregulation and AKT pathway over-activation [36]. One benefit of combination therapy is its potential to counteract this acquired resistance. In this case, co-administration of anlotinib would block upregulated PDGFR and FGFR which are off-target resistance built by eribulin. In addition, CAFs can support cancer cells and help them build resistance [37, 38]. We noticed that CAFs were reduced by combination therapy, which would also impede the tumor from developing resistance. Apart from direct inhibition of tumor cells, anlotinib can also block VEGFR1-3 on endothelial cells and exhibit an anti-angiogenesis effect. We have validated a decrease in MVD after anlotinib treatment in PDX, and our team earlier revealed that MVD is an independent risk factor for overall survival [26]. Given that eribulin can promote intussusceptive angiogenesis and improve tumor perfusion [39, 40], patients may benefit from the MVD decrement induced by combination therapy. These findings may explain why the combination of these drugs can result in a durable response.

Conventional chemotherapy, while effective, may induce a pro-tumor immune microenvironment under certain circumstances. Prior investigations have elucidated that cytotoxic agents such as doxorubicin, carboplatin, and paclitaxel can upregulate PD-L1 expression in cancer cells [41, 42]. In the present study, our observations indicate that eribulin could induce upregulation of PD-L1 expression in RLPS cells. Intriguingly, co-administration of anlotinib effectively mitigates this immune-suppressive milieu. Other researchers have also noticed that the inclusion of TKI drugs counteracts the overexpression of PD-L1 induced by chemotherapy [43]. It is noteworthy that prolonged and excessive ERS has the potential to incite immunogenic cell death (ICD) and expose intrinsic cellular components. Immune cells can recognize those damage-associated molecular patterns (DAMPs) and establish an anti-tumor immune microenvironment [44]. In the LEADER study, a combination of lenvatinib and eribulin increased dendritic cell infiltration in soft tissue sarcoma [24], providing evidence that eribulin combined with TKIs might improve the immune milieu. Collectively, our findings suggest that the proposed combination regimen may facilitate the modulation of the tumor immune microenvironment from a pro-tumor state to an anti-tumor state.

In conclusion, we extensively explored the effective antitumoral effects of the combination of anlotinib and eribulin on RLPS cells both in vivo and in vitro. Indeed, the remarkable efficiency of their combination in affecting cell viability by eliciting continuous ERS may have important implications in the treatment of RLPS.

## Materials and methods

### Primary DDLPS cell strain isolation

Fresh surgical DDLPS samples were rinsed in PBS and minced under sterile conditions with 0.5 mg/ml collagenase type IV and 0.5 mg/ml dispase II. Isolated cells were cultured in A-DMEM/F12 medium containing 15% FBS. Optional Matrigel (356237, Corning, NY, USA) was supplemented during the first few passages. Consecutively passaging cells beyond 30 times is deemed to be a usable cell strain and a total of 3 DDLPS cell strains were established, denoted as DLPS01, DLPS02, and DLPS03 in this article. DLPS02, widely used in this research, was isolated from a 65-year-old female with recurrent retroperitoneal sarcoma, post-operational pathological examination confirmed DDLPS subtype.

### Cell culture

DLPS01, DLPS02, and DLPS03 were routinely cultured in DMEM/F12 medium supplemented with 15% FBS. 93T449, 94T778, and Sw872 cell lines were acquired from the American Type Culture Collection, and routinely cultured in RPMI 1640 medium supplemented with 10% FBS. Cell cultures were maintained in a 37 °C humidified incubator under a 5% CO_2_ environment. DLPS02 cell strain was validated to be contamination-free by short tandem repeat (STR) authentication (Supplementary Fig. 4).

### Organoid establishment and culture

Single cell suspension was acquired as mentioned in primary cell strain isolation, and optional red blood cell lysis was performed. After brief chilling on ice, the cell suspension was mixed with Matrigel and embedded in 24-well plates. Then the plate was inverted and placed in the incubator, allowing the Matrigel dome to solidify. AMMS-MSC medium (AS-13, T&L Biotechnology, Beijing, China) was used to culture DDLPS organoids.

### Reagents

Anlotinib dihydrochloride was manufactured from Selleck Chemical (Houston, TX, USA), and eribulin mesylate was manufactured from Eisai (Hertfordshire, UK). Salubrinal was purchased from MedChemExpress (Monmouth Junction, NJ, USA). Antibodies against CDK4 (12790T), PPAR-γ (2435S), E-cadherin (3195S), N-cadherin (13116P), vimentin (5741P), p-PERK (3179S), Bcl-2 (4233 S), caspase-3 (9662S), PD-L1 (13684S), β-actin (4970S), VEGFR-2 (9698S), FGFR1 (9740S), CD31 (3528S) and GAPDH (5174S) were purchased from Cell Signaling Technology (Beverly, MA, USA). Antibodies against GRP78 (ab21685), PDGFRα (ab203491), CD34 (ab81289), MDM2 (ab16895), Ki-67 (ab16667) and α-SMA (ab7819) were purchased from Abcam (Cambridge, UK). Anti-CHOP antibody was purchased from Abclonal (WH350478, Wuhan, China).

### Cell viability assay

Cells were seeded in 96-well plates with appropriate density and treated with different conditions. At the endpoint, supernatant was aspirated and medium with 10% CCK-8 was added. After 2–3 h of incubation, 450 nm absorbance was measured by a microplate reader, and the viability rate was calculated according to the manufacturer’s guide.

### Colony formation assay

RLPS cells were initially plated in 6-well plates, allowing them to adhere overnight. Then drugs at different concentrations were added. After 14–21 days of culturing, cells were fixed with 4% paraformaldehyde and stained with 0.2% crystal violet solution. Colony number and area were measured utilizing ImageJ (version 1.53k, US National Institutes of Health) under a certain threshold.

### Migration assay

After treatment with anlotinib and eribulin, RLPS cells were trypsinized and collected. They were inoculated in pre-hydrated Transwell chambers (3422, Corning) at a certain cell number. Lower chambers were added medium containing 20% FBS, while upper chambers were added FBS-free medium. After 24 h, chambers were rinsed and stained with 0.2% crystal violet.

### Cell apoptosis and cell-cycle analysis

For apoptosis analysis, propidium iodide (PI) and annexin-FITC (AD10, Dojindo, Kumamoto, Japan) were used to label cells according to the manufacturer’s guide. For cell-cycle analysis, cells were fixed with pre-cold ethanol overnight, and stained with PI/RNase staining buffer (550825, BD, San Diego, CA, USA). All stained cells were assessed by flow cytometer (CytoFLEX, Beckman Coulter, Brea, CA, USA). Cell-cycle data was further analyzed in ModFit (version 4.1, Verity Software House, Bedford, MA, USA).

### RNA-seq and transcriptome analysis

93T449, 94T778, and Sw872 cells were treated with anlotinib and eribulin for 24 h, at which concentration reached the maximum synergy score. Cells were then rinsed and lysed by TRIzol reagent. RNA extraction and following sequencing were conducted by Beijing Mygenostics Co. Ltd. In brief, mRNA was enriched by polyA tail, and RNA integrity was checked by Bioanalyzer 2100 system, then cDNA was synthesized and purified. Quality-checked library was sequenced on the Illumina Novaseq platform, and further reads quality control, and gene mapping were performed based on general industrial standards. Differential expression was analyzed through the DESeq2 R package using the pairwise method.

### Protein extraction and western blot

Differently treated cell cultures were washed with PBS and lysed by strong RIPA buffer mixed with protease and phosphatase inhibitors. Tissues from xenograft were homogenized with RIPA buffer using a homogenizer (KZ-III-F, Servicebio, Wuhan, China). The lysate was sequentially centrifuged and the supernatant protein concentration was quantified by BCA assays. After standard SDS-PAGE and blotting, PVDF membranes were blocked by 5% skimmed milk and probed with antibodies. Thereafter, washed membranes were visualized by chemiluminescence. Cancer-associated fibroblast lysate was previously extracted and preserved [45].

### Patient-derived xenograft (PDX) establishment and animal experiment

In this study, 6-week-old NOD/SCID mice were used and housed in an SPF-level facility. Primary PDX originated from a 59-year-old male patient with recurrent retroperitoneal liposarcoma and was pathologically diagnosed as DDLPS after surgery. A 20–30 mm^3^ tumor piece was implanted at one flank of a mouse. Once the PDX grew to about 1000 mm^3^, the tumor-bearing mouse was sacrificed, and the tumor was passed to another mouse. For experimental purposes, mice at passage 4 were used. Tumors from passage 3 were dissected into small pieces and transplanted into 30 mice for further experimentation. Upon reaching a tumor volume of 100–150 mm^3^, the mice were randomly assigned to six groups, and drug administration commenced. Tumor size was measured every 3 days until reaching the humane endpoint or euthanasia. Tumor volume is estimated using the formula *V* = 0.5 × length × width^2^.

### Immunochemistry staining (IHC) and fluorescence in-situ hybridization (FISH) staining

Slices were deparaffinized and rehydrated through a series of xylene and ethanol, then treated in 3% hydrogen peroxide. Antigen retrieval was performed based on antibody-specific instruction. After the first and second antibody incubation, DAB was used to detect protein expression. A fluorophore-labeled MDM2 probe was used to detect chromosome 12q13–q15 amplification according to the manufacturer’s protocol (JLB301002, Jinlu Bio, Shaoxing, China).

### Statistical and data analysis

The GSE21122 dataset was acquired from the Gene Expression Omnibus (GEO) database (https://www.ncbi.nlm.nih.gov/geo/query/acc.cgi?acc=GSE21122). Dose-response curves and IC_50_ values were calculated by GraphPad Prism (Version 9.5.0, GraphPad Software, San Diego, CA, USA) with a 4-parameter setting. Synergy heatmaps were generated by the SynergyFinder R package, employing the zero interaction potency (ZIP) model [46]. Unless otherwise specified, data with individual replicates are presented as the means ± standard deviation (SD) and significance was tested by unpaired two-tailed Student’s *t*-test. Detailed bioinformatics methods, software, and R packages are listed in Supplementary Table 1.

### Supplementary information

SUPPLEMENTARY MATERIAL

Original Western Blot Data

## Data Availability

The raw and processed transcriptome data are available in the GEO database (GSE261076). The data and materials in this study are available from the corresponding author upon reasonable request.
