# Supplementary material for: Combination of eribulin and anlotinib exerts synergistic cytotoxicity in retroperitoneal liposarcoma by inducing endoplasmic reticulum stress
Source: Cell Death Discov. 2024 Aug 8;10:355. doi: 10.1038/s41420-024-02103-2 (PMC11310505; doi:10.1038/s41420-024-02103-2)
Supplement: Supplementary file 1 — SUPPLEMENTARY MATERIAL [file 41420_2024_2103_MOESM1_ESM.pdf]

SUPPLEMENTARY MATERIAL FOR

Combination of Eribulin and Anlotinib Exerts Synergistic Cytotoxicity in Retroperitoneal Liposarcoma by Inducing Endoplasmic Reticulum Stress

Shuquan Li [1], Hongtao Zhang [2], Hao Yu [1], Yifan Wu [1], Liang Yan [1], Xiaoya Guan [1], Bin Dong [3], Min Zhao [4], Xiuyun Tian [1*], Chunyi Hao [1*], Jianhui Wu [1*]

1. Key Laboratory of Carcinogenesis and Translational Research (Ministry of Education), Department of Hepato-Pancreato-Biliary Surgery, Peking University Cancer Hospital & Institute, Beijing, China.
2. Guowen (Changchun) International Hospital, Jilin Province, China.
3. Key Laboratory of Carcinogenesis and Translational Research (Ministry of Education), Central Laboratory, Peking University Cancer Hospital & Institute, Beijing, China.
4. Key Laboratory of Carcinogenesis and Translational Research (Ministry of Education), Department of Pathology, Peking University Cancer Hospital & Institute, Beijing, China.

[*]Corresponding Authors:
Jianhui Wu, E-mail: wujianhui@bjmu.edu.cn
Chunyi Hao, E-mail: haochunyi@bjmu.edu.cn
Xiuyun Tian, E-mail: tianxiuyun@bjmu.edu.cn

This document contains:

- Supplementary Figure 1. Transcriptome analysis of combination of anlotinib and eribulin
- Supplementary Figure 2. *In vivo* study of anlotinib and eribulin combination
- Supplementary Figure 3. Combination regimen modulated TME in RLPS patients
- Supplementary Figure 4. STR profile of DLPS02 cell strain
- Supplemental Table 1. Bioinformatics software/ R packages used in this study

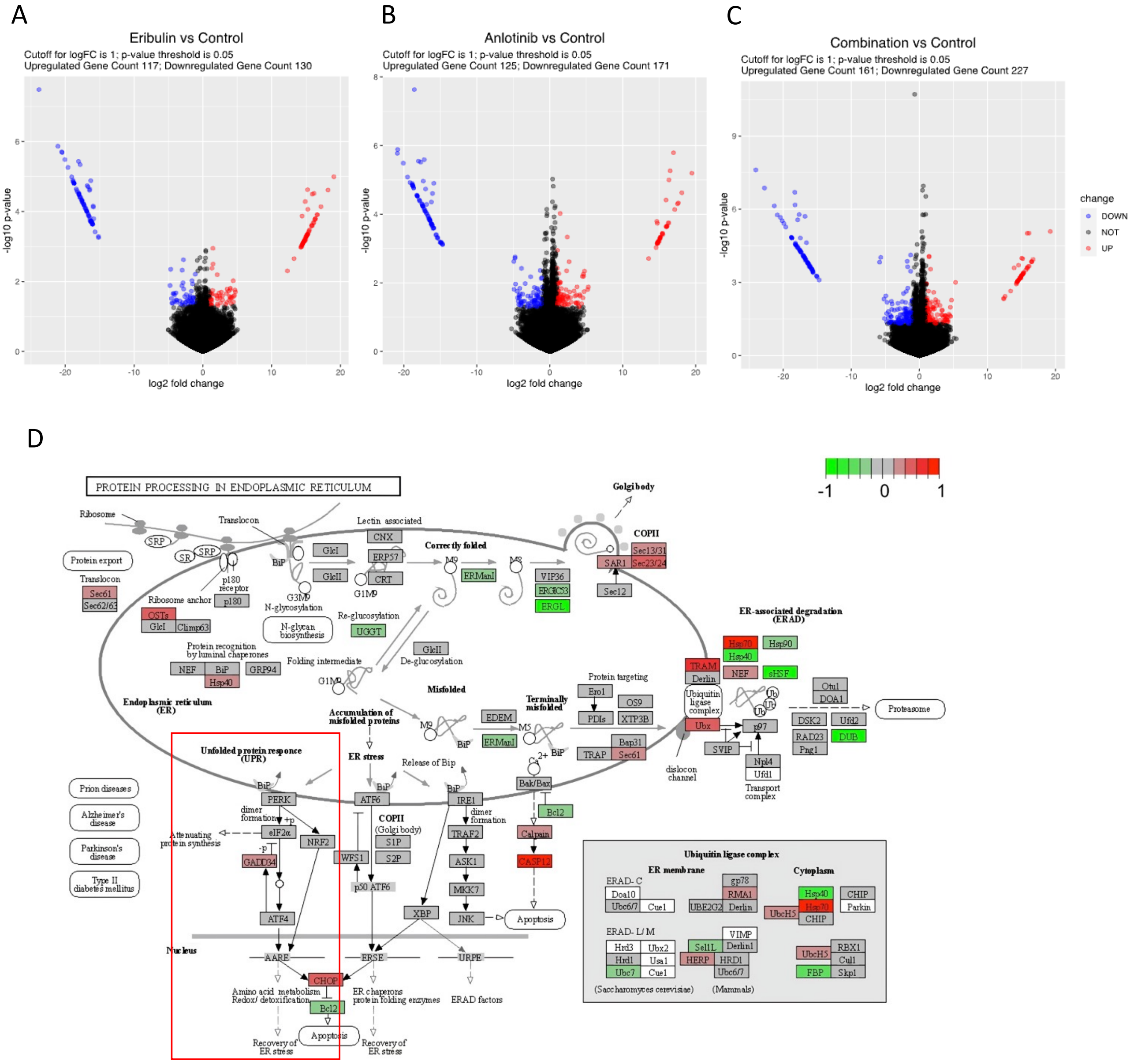

Supplementary Figure 1. Transcriptome analysis of combination of anlotinib and eribulin

A-C, Volcano plot of anlotinib, eribulin, combination group comparing to control group. D, Detailed pathway analysis of protein processing in endoplasmic reticulum. Red box showing PERK related pathway in endoplasmic reticulum stress.

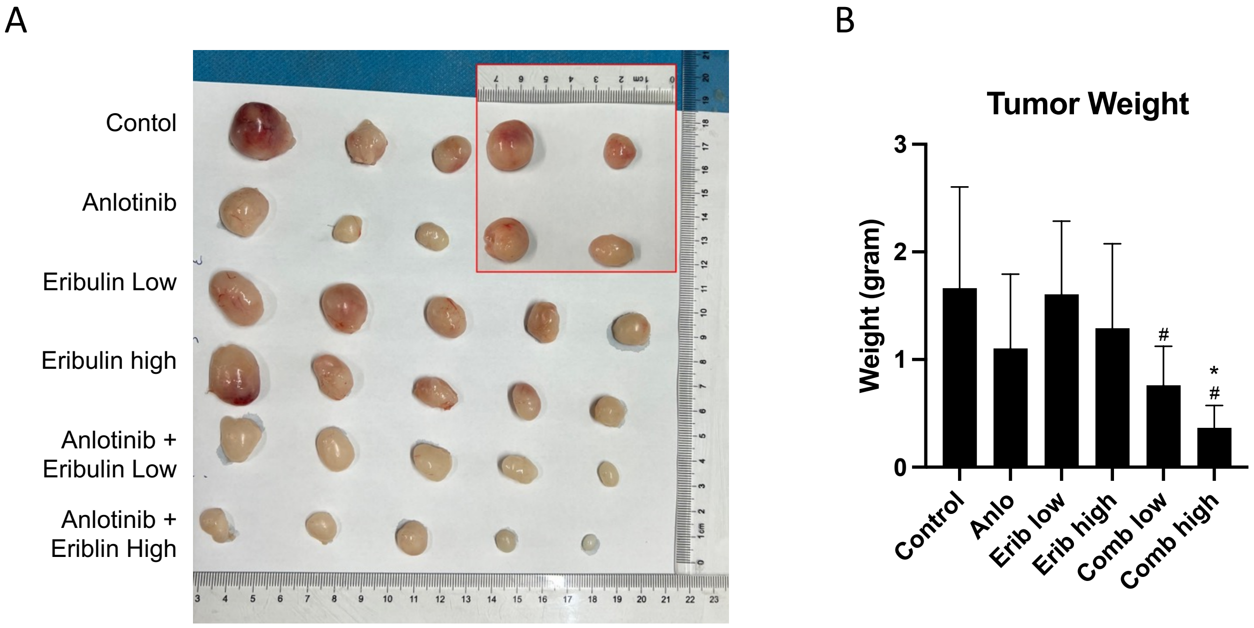

Supplementary Figure 2. *In vivo* study of anlotinib and eribulin combination

A, Macroscopic examination of RLPS PDX in each group. Tumor in red box is sacrificed in different batch, because they did not meet 100-150 mm^3 size at start of administration. ALL 30 PDXs showing in this picture were in the same passage number and treated exactly the same. B, Tumor weight for each group. $^{\#}$ compared to eribulin group (with corresponding concentration), $^{\#}$/*p < 0.05.

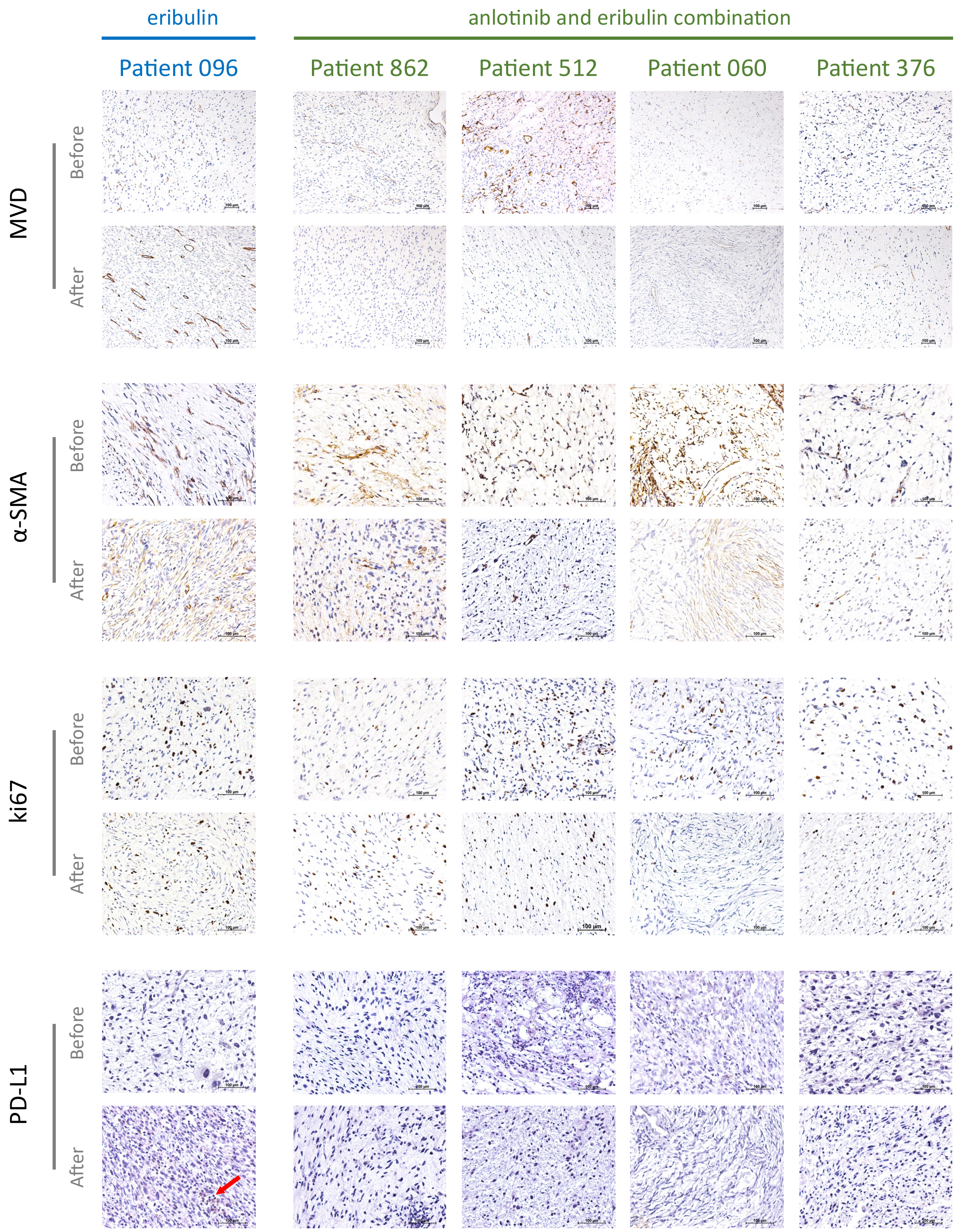

Supplementary Figure 3. Combination regimen modulated TME in RLPS patients

A total 5 RLPS patients received surgery before and after eribulin (n=1) or combination (n=4) treatment. Paired samples were collected and conducted IHC staining. CD31 labeled micro-vessels and α-SMA labeled CAFs were decreased in combination group comparing to eribulin treated patient. Ki-67 is an indicator for proliferation, and it was reduced in combination group. Due to relative low expression rate of PD-L1, PD-L1 expression was not observed before and after combination treatment. However, after eribulin treatment, clusters of cancer cells expressing PD-L1 could be observed (indicated by red arrow). MVD: micro-vessel density.

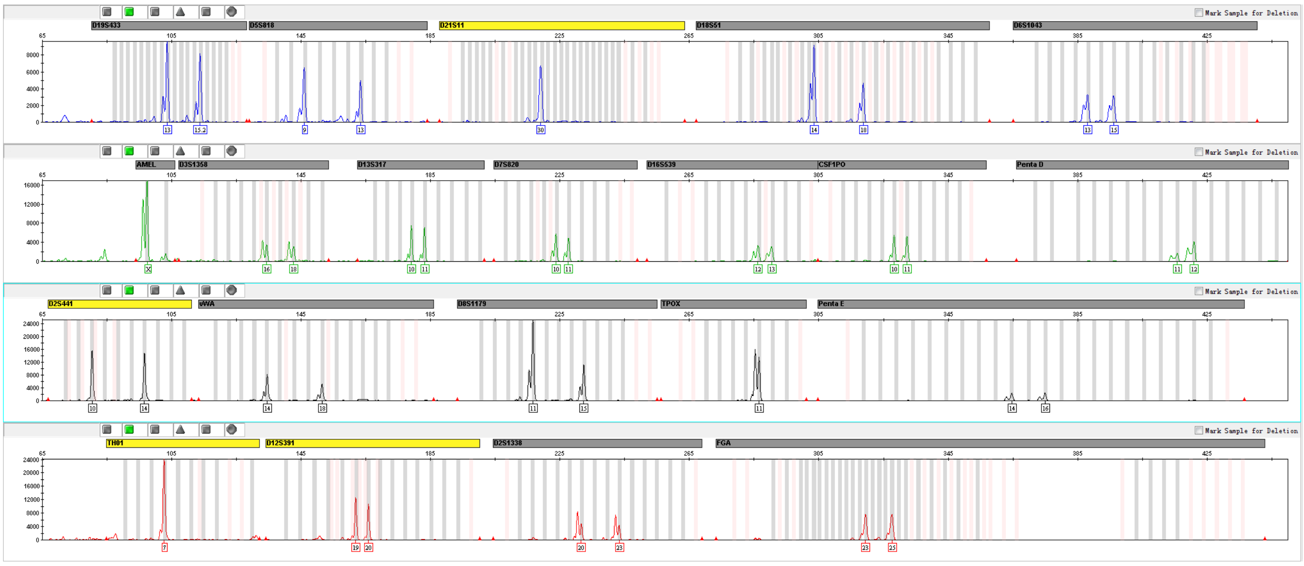

Supplementary Figure 4. STR profile of DLPS02 cell strain

DNA of DLPS02 was amplified with STR site specific primers (Microreader TM 21 ID System) and further processed by genetic analyzer (3130xl, Applied Biosystems). STR profile of DLPS02 was compared with established cell line database (American Type Culture Collection and German Collection of Microorganisms and Cell Cultures), and no matches were found. DLPS02 was proved to be human derived cell with no contamination of other cell lines.

Software / Package	Version	Description
fastp	0.23.0	Raw RNAseq data quality control
R	4.2.1 (8095)	Platform for analysis
RStudio	2023.03.0+386	Platform for analysis
clusterProfiler	4.7.1.003	Enrichment tool for omics data
DESeq2	1.36.0	Differential gene expression analysis
enrichplot	1.18.3	Enrichment results visualization
ggplot2	3.4.3	Data visualization
pathview	1.36.1	Pathway alteration visualization
synergyfinder	3.4.5	Synergy score calculation and visualization

Supplemental Table 1. Bioinformatics software/ R packages used in this study
