# Supplementary material for: Combination of eribulin and anlotinib exerts synergistic cytotoxicity in retroperitoneal liposarcoma by inducing endoplasmic reticulum stress
Source: Cell Death Discov. 2024 Aug 8;10:355. doi: 10.1038/s41420-024-02103-2 (PMC11310505; doi:10.1038/s41420-024-02103-2)
Supplement: Supplementary file 2 — Original Western Blot Data [file 41420_2024_2103_MOESM2_ESM.docx]

# Original Western Blot Data

In this study, protein ladder marker #26616 (Thermo Scientific) and RM19001 (Abclonal) was used. Detailed marker layout could be found on <https://www.thermofisher.com/order/catalog/product/26616> and <https://abclonal.com/molecular-biology/ColorMixed-Protein-Marker-180/RM19001>. Here we list all raw data of merged image, showing prestained protein marker with molecular weight.

## Figure 2C

4-12% gradient gel was used.

| CDK4 @30kd | 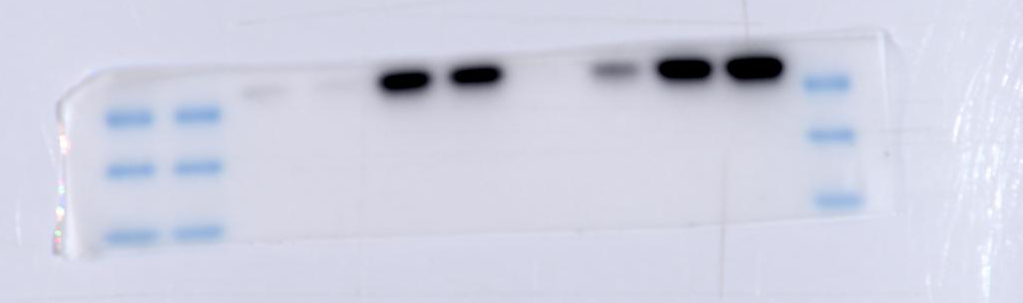 |
| --- | --- |
| MDM2 @90kd | 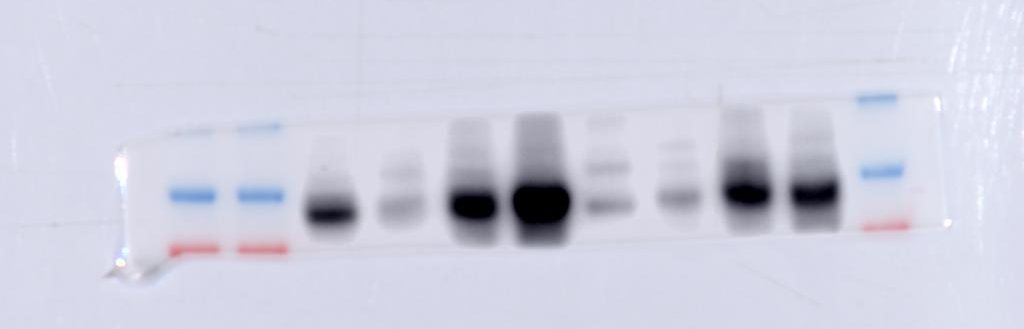 |
| PPAR-gamma @53&57kd | 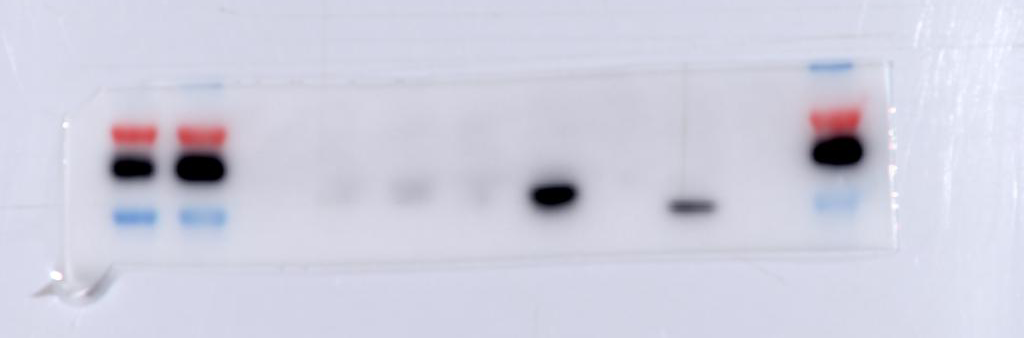 |
| a-SMA @42kd | 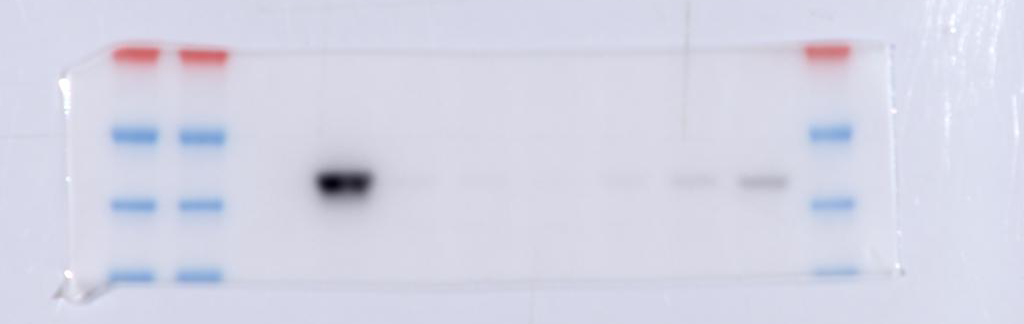 |
| E-cadherin @135kd | 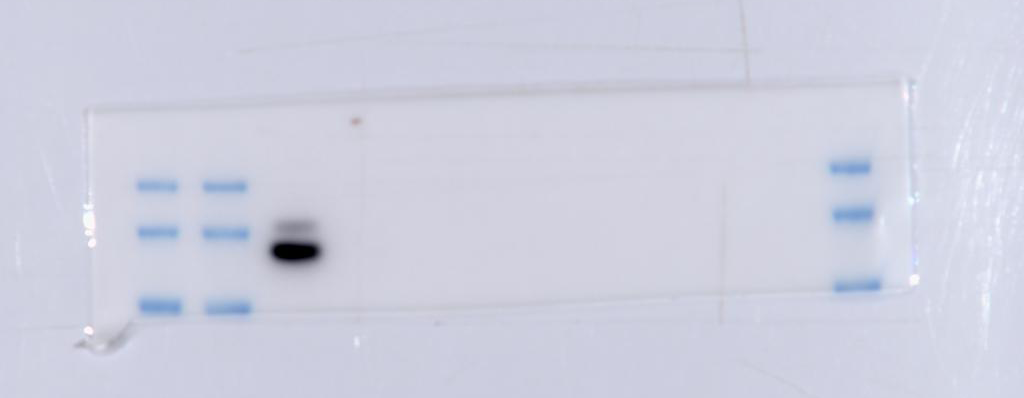 |
| N-cadherin @140kd | 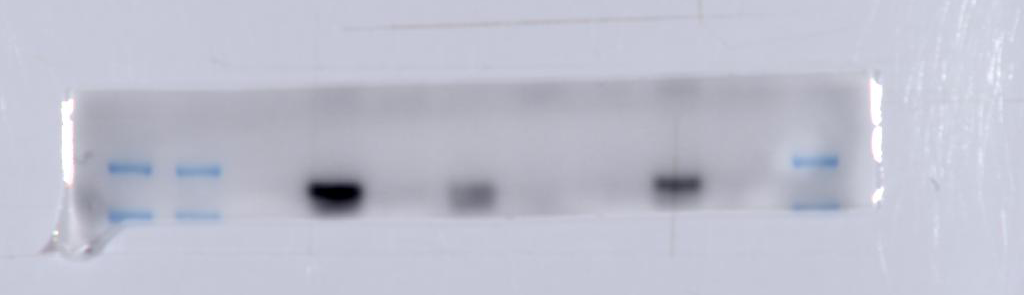 |
| Viementin @57kd | 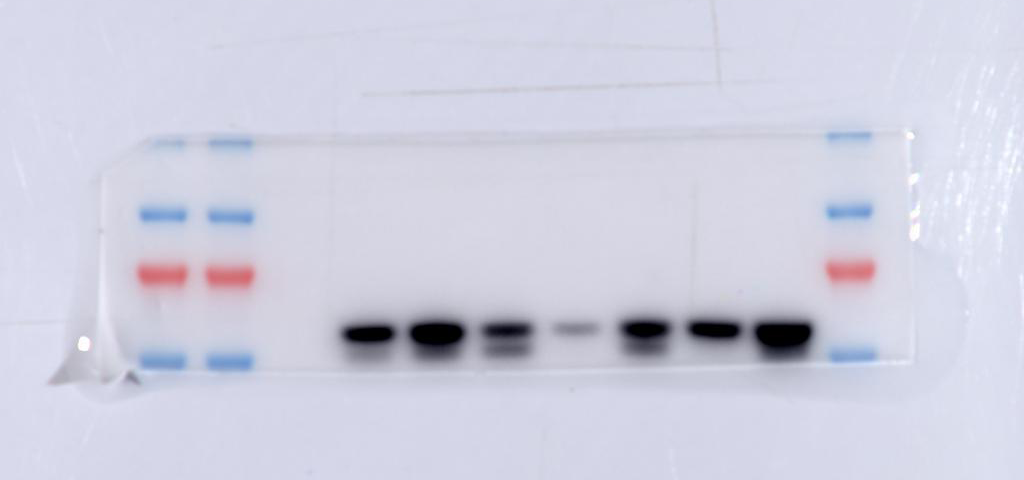 |
| GAPDH @36kd | 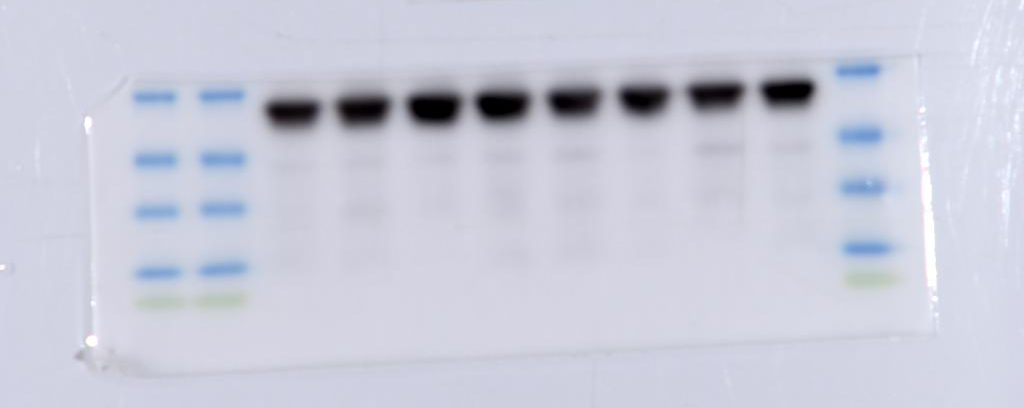 |

## Figure 5E 94T778

10% gel was used.

| GRP78 @78kd | 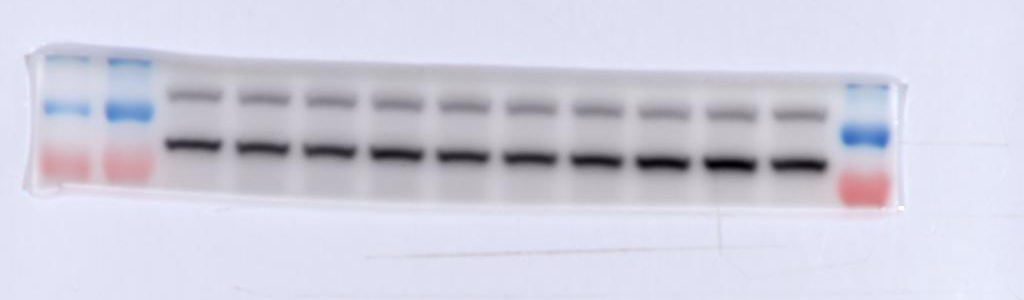 |
| --- | --- |
| p-PERK @170kd | 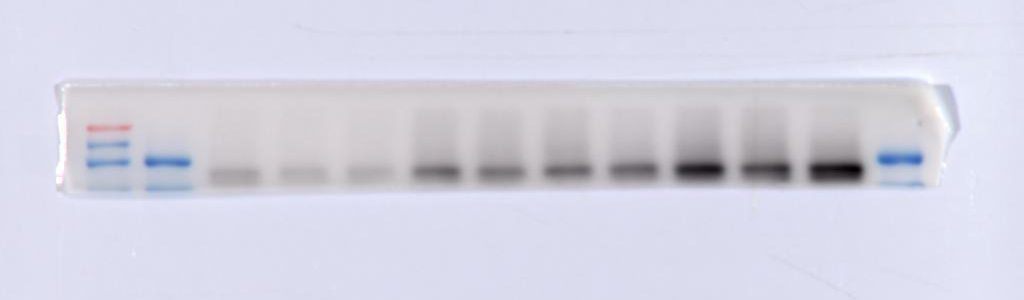 |
| CHOP @30kd | 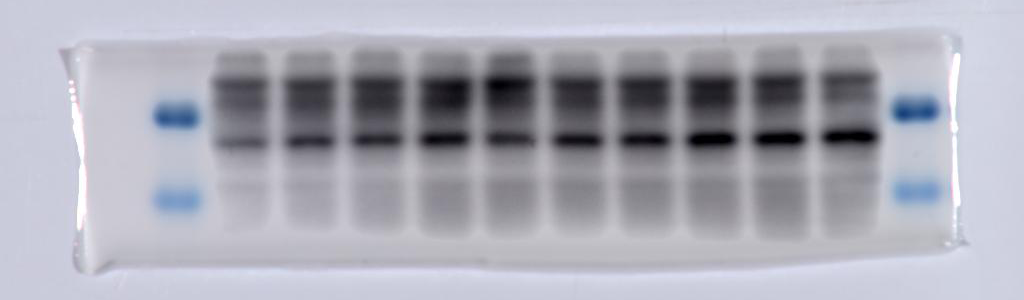 |
| Bcl2 @26kd | 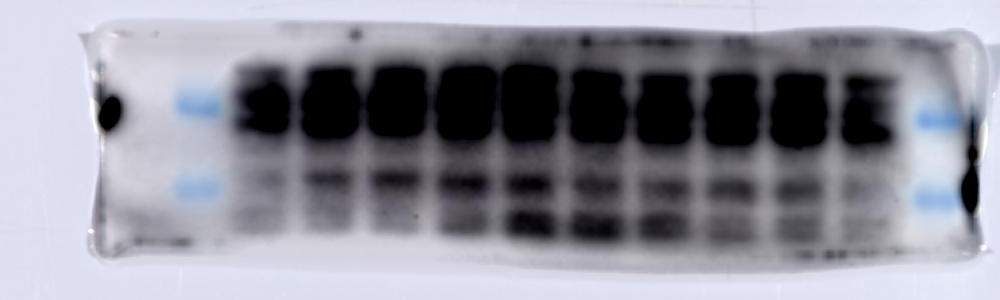 |
| Caspase3 @~15-19kd | 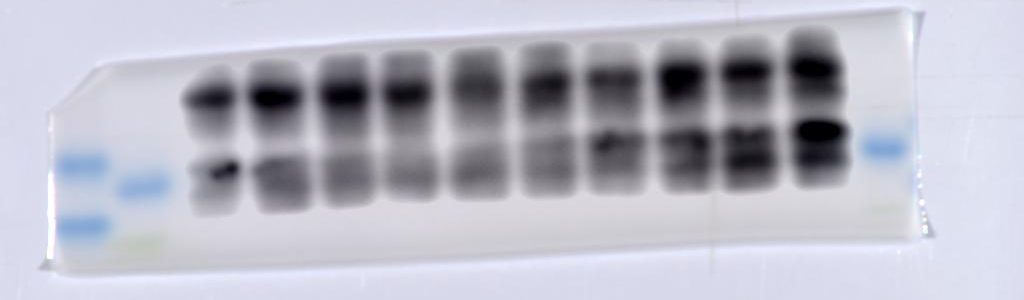 |
| PD-L1 @~40-50kd | 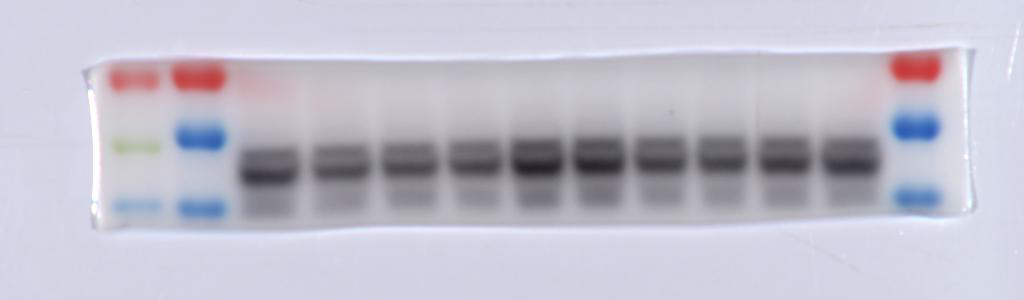 |
| Beta-actin @42kd | 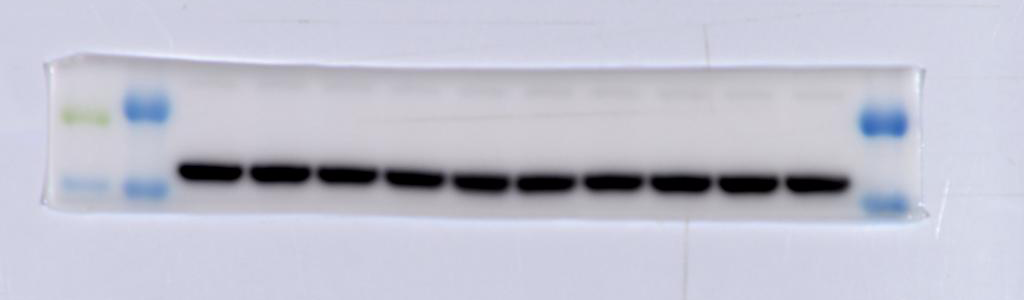 |

## Figure 5E DLPS02

10% gel was used.

| Grp78 @78kd | 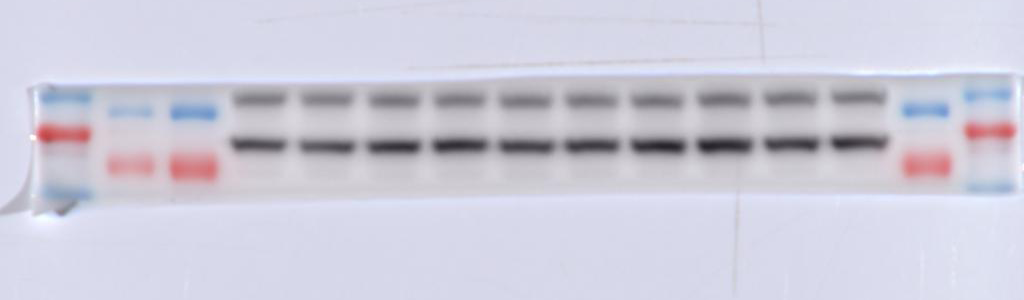 |
| --- | --- |
| p-perk @170kd | 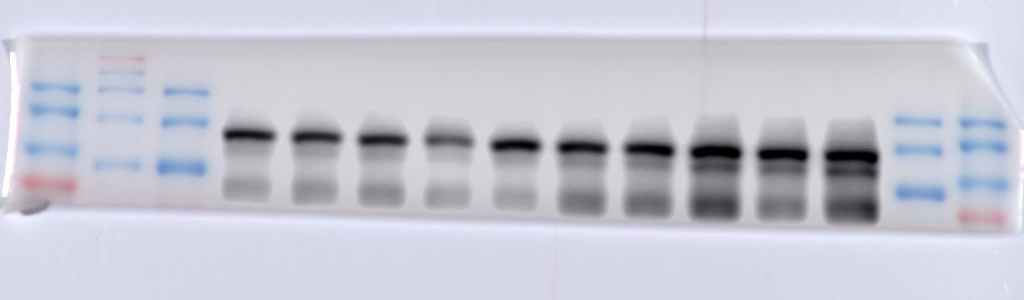 |
| CHOP @30kd | 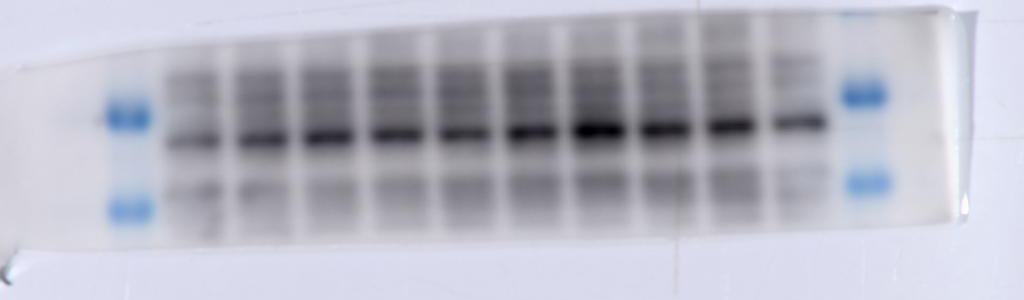 |
| Bcl2 @26kd | 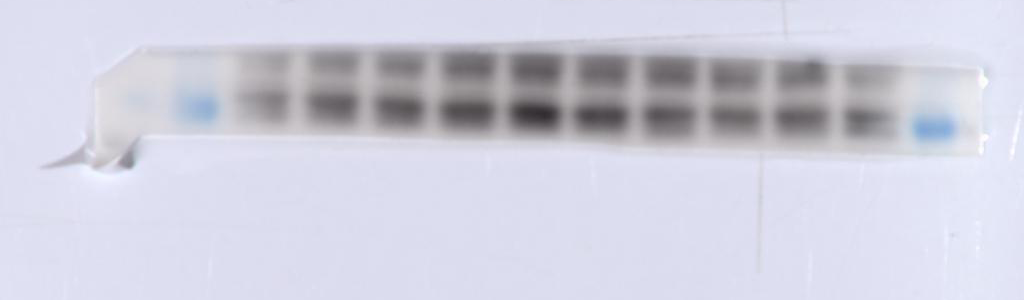 |
| Caspase3 @~15-19kd | 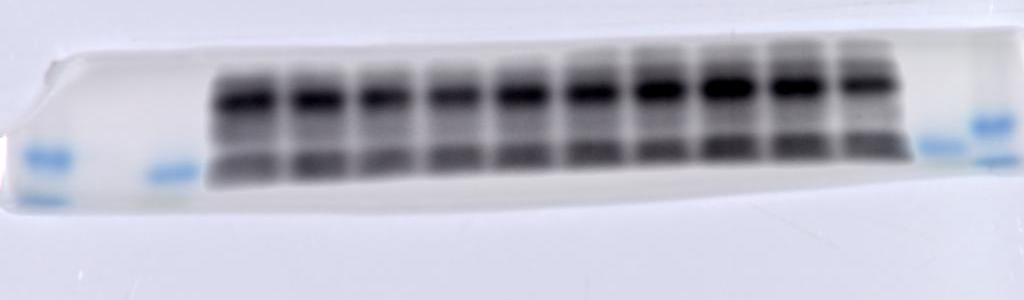 |
| PD-L1 @~40-50kd | 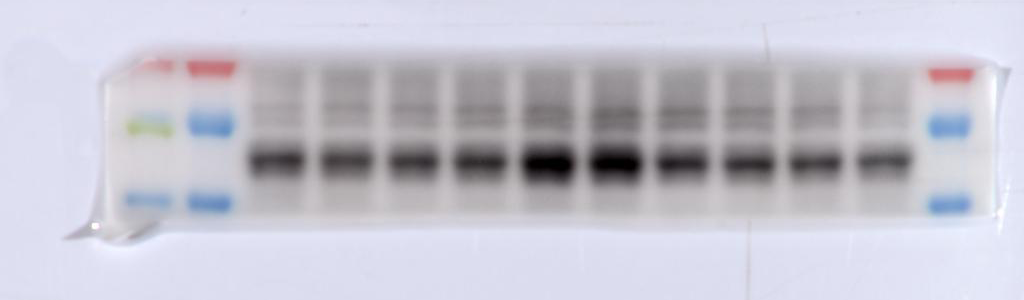 |
| Beta-actin @42kd | 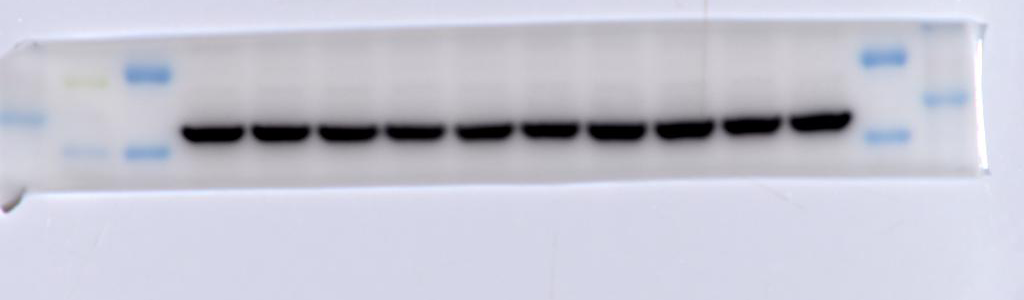 |

## Figure 5E Sw872

10% gel was used.

| Grp78 @78kd | 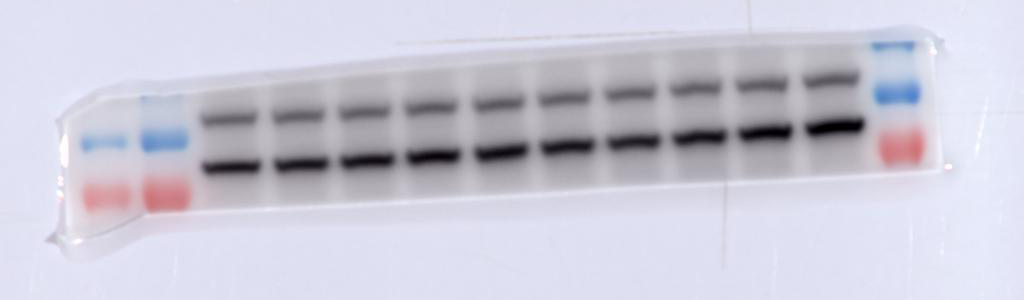 |
| --- | --- |
| p-perk @170kd | 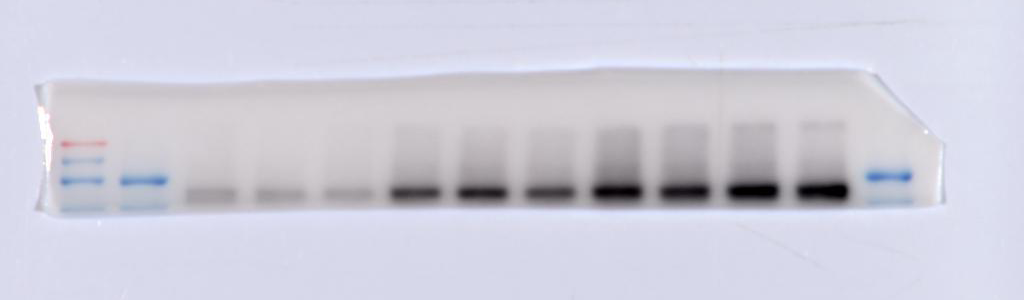 |
| CHOP @30kd | 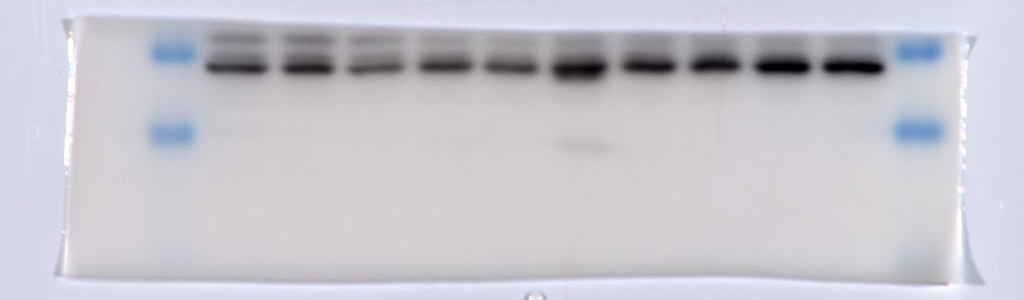 |
| Bcl2 @26kd | 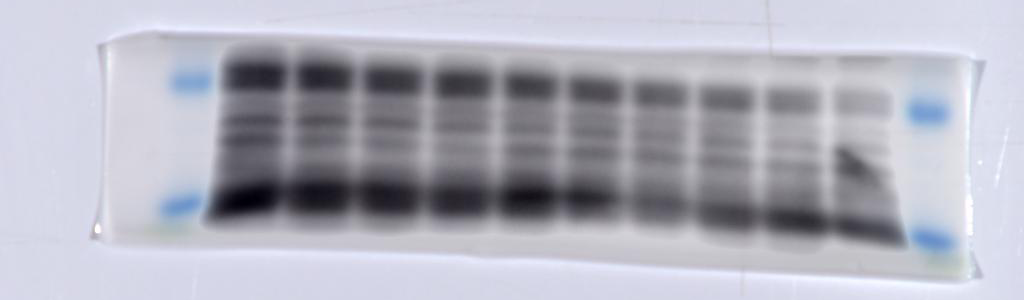 |
| Caspase3 @~15-19kd | 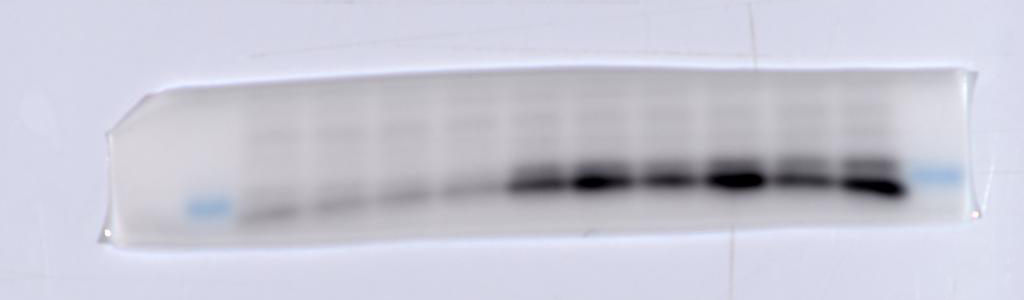 |
| PD-L1 @~40-50kd | 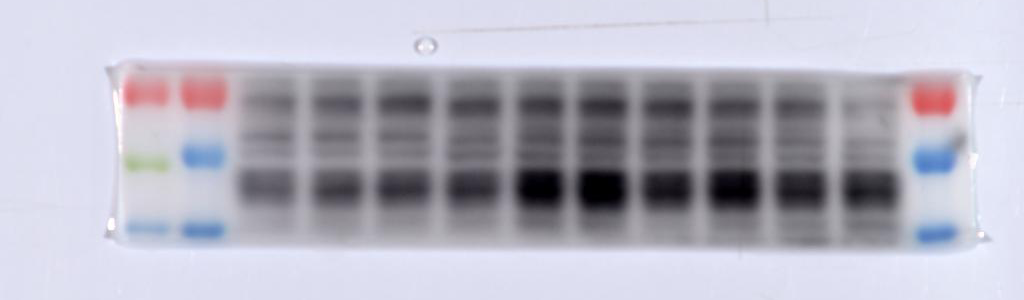 |
| Beta-actin @42kd | 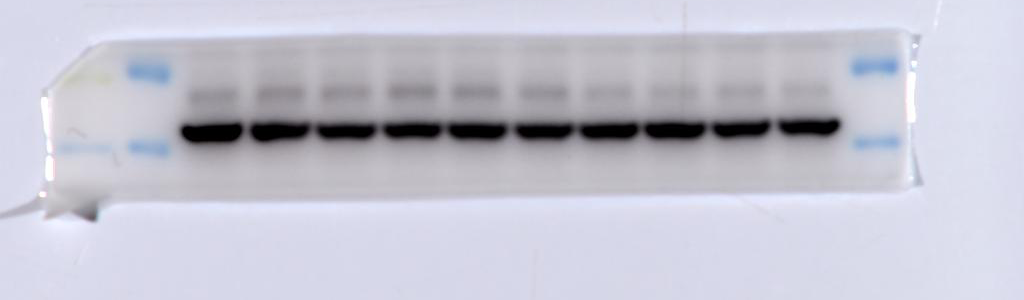 |

## Figure 6D

10% gel was used.

| VEGFR2 @ 230kd | 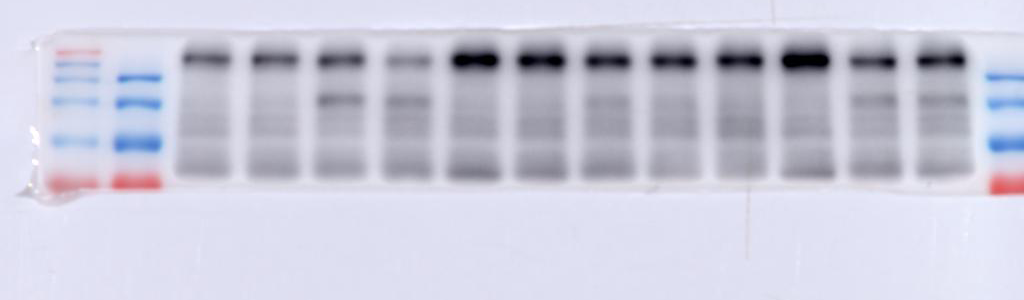 |
| --- | --- |
| PDGFR-a @190kd | 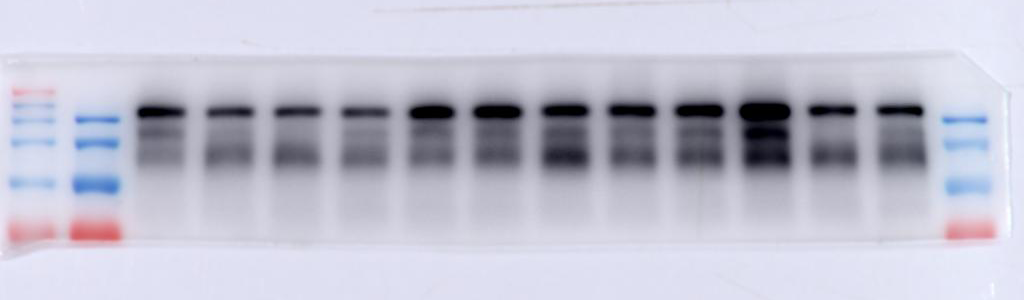 |
| FGFR1 @120-145kd | 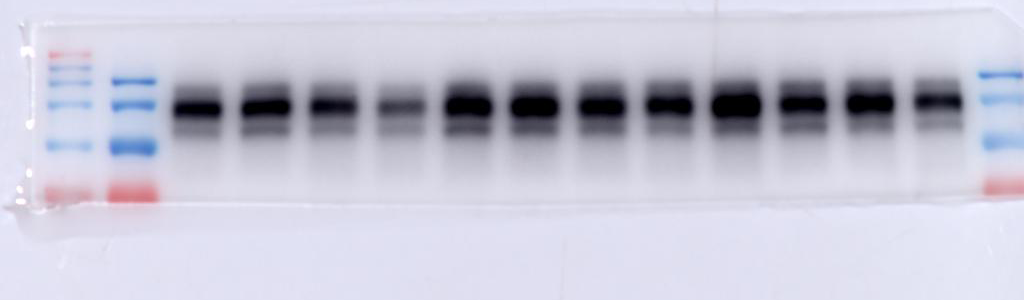 |
| PD-L1 @~40-50kd | 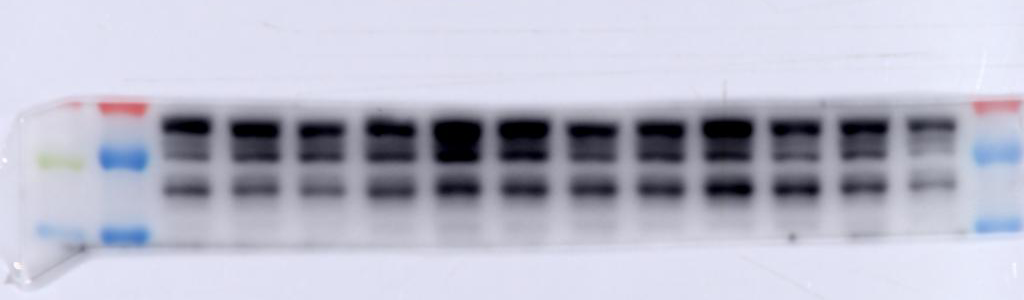 |
| Gapdh @36kd | 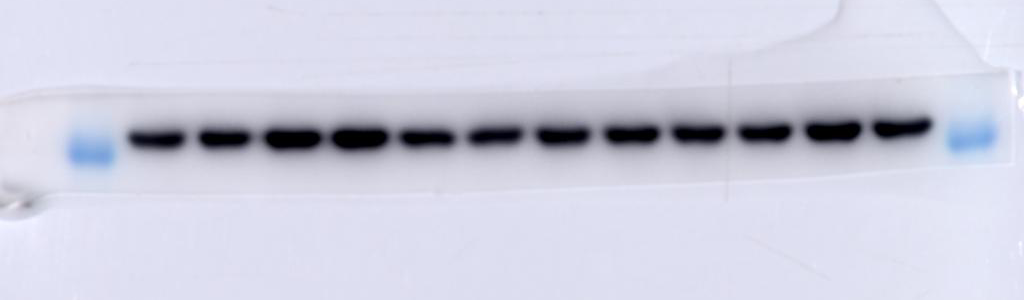 |

## Figure 6E

10% gel was used.

| CHOP @30kd | 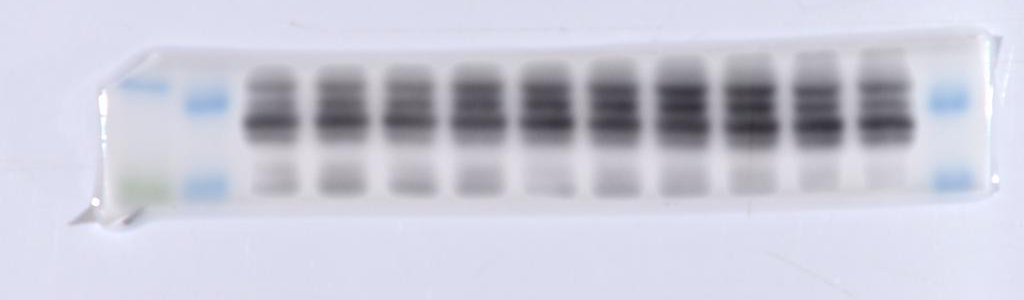 |
| --- | --- |
| Beta-actin @42kd | 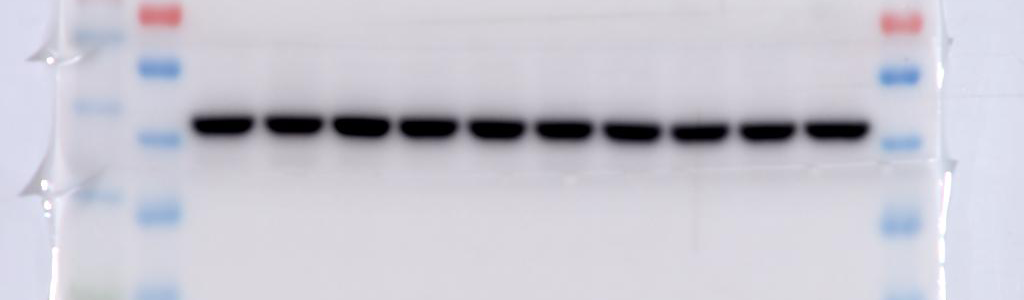 |
